# Altered Patterns of Gene Expression Underlying the Enhanced Immunogenicity of Radiation-Attenuated Schistosomes

**DOI:** 10.1371/journal.pntd.0000240

**Published:** 2008-05-21

**Authors:** Gary P. Dillon, Theresa Feltwell, Jason Skelton, Patricia S. Coulson, R. Alan Wilson, Alasdair C. Ivens

**Affiliations:** 1 Department of Biology, University of York, York, United Kingdom; 2 Pathogen Microarrays Group, Wellcome Trust Sanger Institute, Hinxton, United Kingdom; Queensland Institute of Medical Research, Australia

## Abstract

**Background:**

Schistosome cercariae only elicit high levels of protective immunity against a challenge infection if they are optimally attenuated by exposure to ionising radiation that truncates their migration in the lungs. However, the underlying molecular mechanisms responsible for the altered phenotype of the irradiated parasite that primes for protection have yet to be identified.

**Methodology/Principal Findings:**

We have used a custom microarray comprising probes derived from lung-stage parasites to compare patterns of gene expression in schistosomula derived from normal and irradiated cercariae. These were transformed *in vitro* and cultured for four, seven, and ten days to correspond in development to the priming parasites, before RNA extraction. At these late times after the radiation insult, transcript suppression was the principal feature of the irradiated larvae. Individual gene analysis revealed that only seven were significantly down-regulated in the irradiated versus normal larvae at the three time-points; notably, four of the protein products are present in the tegument or associated with its membranes, perhaps indicating a perturbed function. Grouping of transcripts using Gene Ontology (GO) and subsequent Gene Set Enrichment Analysis (GSEA) proved more informative in teasing out subtle differences. Deficiencies in signalling pathways involving G-protein–coupled receptors suggest the parasite is less able to sense its environment. Reduction of cytoskeleton transcripts could indicate compromised structure which, coupled with a paucity of neuroreceptor transcripts, may mean the parasite is also unable to respond correctly to external stimuli.

**Conclusions/Significance:**

The transcriptional differences observed are concordant with the known extended transit of attenuated parasites through skin-draining lymph nodes and the lungs: prolonged priming of the immune system by the parasite, rather than over-expression of novel antigens, could thus explain the efficacy of the irradiated vaccine.

## Author Summary

*Schistosoma mansoni* is a blood-dwelling parasitic worm that causes schistosomiasis in humans throughout Africa and parts of South America. A vaccine would enhance attempts to control and eradicate the disease that currently relies on treatment with a single drug. Although a manufactured vaccine has yet to generate high levels of protection, this can be achieved with infective parasite larvae that have been disabled by exposure to radiation. How these weakened parasites are able to induce protective immunity when normal parasites do not, is the question addressed by our experiments. We have used a technique of gene expression profiling to compare the patterns in normal and disabled parasites, over the period when they would trigger an immune response in the host. We found that only a handful of genes were differentially expressed, all of them diminished in the disabled parasite. However, a more sensitive technique to examine groups of genes revealed that those involved in nervous system and muscle function were depressed in the disabled parasites. We suggest that reduced mobility of these larvae permits them longer contact with the immune system, thus enabling a strong protective immune response to develop.

## Introduction

The radiation-attenuated schistosome (RA) vaccine remains the most effective way of inducing high levels of protective immunity against *Schistosoma mansoni* in rodent and primate hosts (reviewed by Coulson) [1]. However, an effective recombinant vaccine based upon it, for use in humans, has thus far proved elusive [2]. Few differences have been reported between irradiated and normal larvae apart from an altered morphological phenotype at the lung stage of development [3] that produced subtle differences in motility. This accorded with a key feature of the vaccine that attenuated larvae must undergo a truncated migration, as far as the lungs, to prime the immune system [4]. Furthermore, extensive parasite tracking [5] and immunological investigations [6] have revealed the lung schistosomulum to be the principal target of immune effector responses in the murine host. The requirement for CD4^+^ T cells [7] means that antigens must be released by, or exposed on, the surface of target larvae for processing and presentation by accessory cells to trigger such effector responses.

Targets of protective immunity have historically been identified by screening crude antigen preparations and expression libraries with sera from putatively immune hosts [8],[9]. In the schistosome context such screens have, in the main, produced a catalogue of abundant cytoplasmic proteins that one would not ordinarily expect to be secreted or surface-exposed and thus available to the immune system. Indeed, the abundance and antigenicity of cytoplasmic proteins appears to pose a major obstacle to identifying truly protective antigens. Abundant transcripts can dominate the content of cDNA libraries; equally, highly expressed proteins may mask attempts to identify *bona fide* vaccine candidates using proteomics [10] or immunoproteomics [11]. Clearly alternative approaches are needed to pinpoint antigens relevant to protection in this model and the sequencing of the schistosome transcriptome [12] and genome (www.GeneDB.org) now provide unparalleled opportunities for rapid progress using post-genomic techniques [13].

We have previously constructed a microarray comprising cDNAs derived from normal lung stage schistosomula and used it to identify genes highly expressed in the migrating parasite relative to six other life cycle stages [14]. We found genes encoding six membrane, six membrane-associated and five secreted proteins that were preferentially expressed at the lung or skin and lung stage. However, when considered in isolation it is difficult to predict which of these proteins, if any, will make suitable vaccine candidates. Their site of expression in the complex parasite body is unknown and some are hypothetical proteins with no ascribed function except at the motif or domain level. We now report use of the same lung stage array to pinpoint transcripts differentially expressed between normal and irradiated parasites cultured to the lung stage. This experiment was designed to identify the molecular changes underlying the altered phenotype, primarily using Gene Set Enrichment Analysis (GSEA) to delineate groups of genes with associated functions, which could explain the enhanced immunogenicity of the irradiated larvae.

## Methods

### Biological material

A Puerto Rican isolate of *S. mansoni* was maintained by passage through NMRI strain mice and *Biomphalaria glabrata* snails, the animal work being approved by the Biology Department Ethics Committee, University of York. The microarray [14] was screened with mRNA from schistosomula, derived from mechanically transformed cercariae and grown *in vitro* for four, seven or ten days [15]. The times were chosen on the basis of previous parasite tracking [4] and lymphadenectomy experiments [16]. Attenuated schistosomula begin to accumulate in the lymph node and lung at day four, reaching a plateau in both sites at day seven [4]. Excision of skin-draining lymph nodes at, or prior to, day ten has a major ablative effect on subsequent protection [16] The cercariae were obtained by exposing snails with a patent infection to a bright light. Prior to culture one half of each cercarial shed was exposed to 200 Gray of radiation from an X-ray source at Cookridge Hospital, Leeds.

### Experimental design

The microarray (ArrayExpress A-SGRP-2/E-TABM-408) containing approximately 6000 features printed in duplicate (accession numbers AM042715-AM048613), the hybridisation protocol, and array scanning were as described in Dillon *et al.* (2006). The array represents 3088 unique sequence contigs and singlets, encompassing an estimated 44% of the lung worm transcriptome [12]. At each of the day four, seven and ten sampling times total RNA was extracted from parallel cultures of normal and irradiated schistosomula with Trizol (Invitrogen) according to manufacturer's instructions. Each total RNA was labelled with Cy3 or Cy5 dyes (Perkin Elmer), without amplification, before hybridisation to the array at 20μg per channel [14]. Analysis of the normal and irradiated treatments, in pairs, at three time points encompassed twelve slides, comprising three biological replicates per treatment and one technical replicate (i.e. one of the biological replicates was split and repeated in order to control for experimental error). Dye swaps were balanced across treatments to limit bias resulting from differential dye incorporation and intensity, i.e. 50% of irradiated samples were labelled with Cy3 and 50% with Cy5. One sample from day ten failed to label so only 3 slides in total contributed to that time point.

### Data analysis

The quantative dataset obtained using the GenePix 4000B instrument (Axon Instruments Inc.), was analysed with the GenePix Pro software and the R language for statistical computing (www.r-project.org) [17]. Specifically, the data was processed with the microarray analysis tools available from the Bioconductor Project, a tool for the analysis and comprehension of genomic data (www.bioconductor.org) [18]. The background was subtracted from array data using a Bayesian model-based method [19]. Array data were normalized using the LIMMA component (Linear Models for Microarray Data) of the Bioconductor package [20] with printtip loess to correct for spatial and other artefacts generated during the printing process. (Loess is a locally weighted polynomial regression; see LIMMA documentation.) Linear models were applied and significance statistics generated using empirical Bayesian methods to assess differential gene expression. This has the effect of borrowing information from the ensemble of genes to aid with inference about each individual gene [20]. An observation was classed as significant if it exceeded a natural log-odds (lods) cutoff of 3. To determine the effects of radiation, irrespective of sampling time, normal and irradiated results were pooled and reanalysed as a two way comparison. Detailed description of the methods used can be found in the LIMMA documentation: http://bioconductor.org/packages/2.1/bioc/vignettes/limma/inst/doc/usersguide.pdf.

### Gene set enrichment analysis (GSEA)

As each EST is duplicated on the array, mean red and green values for the 6528 probes were generated from background-subtracted red and green fluorescence values. The LIMMA function “normalizeQuantiles” *was applied to these mean fluorescence values to normalize between arrays. Thus each quantile of each EST is adjusted to its mean across all arrays, irrespective of channel, normalising the data by ensuring the signal intensities within each treatment have the same empirical distribution. In those instances in which two or more ESTs on the array were members of the same Sm contig, the mean normalised values were taken, resulting in a single value for each contig. The normalised signal intensities were combined into tables containing all Sm contigs and singlets, with their GO/Protein analyst annotation (as described in Dillon *et al.* 2006), test channel signals and reference channel signals, prior to submission to the GSEA package. GSEA statistically assesses whether expression of groups of genes correlates with a given phenotype, and requires those groups to contain 15 or more members to function [21]. It quantifies the enrichment of individual members at the top and bottom of a ranked list of gene expression. The enrichment score (ES) is calculated by parsing the ranked gene list for members of a single category, and increasing a running-sum statistic when one of those genes is encountered or decreasing that sum if it is not. The enrichment score is then normalized by adjusting for the number of genes in a category and the GSEA package estimates the significance of each normalized enrichment score (NES) by calculating a false discovery rate (FDR). Gene sets were deemed to be enriched when the FDR ≤ 0.25, this apparently relaxed cut-off being used because the primary goal of GSEA , as specified by Subramanian *et al.* (2005), is to generate hypotheses rather than exclude every last false positive. The FDR is calculated by comparing the tails of the observed and null distributions for the NES. The null is produced by randomly assigning phenotype labels and producing a reordered gene list; this is done 1000 times to generate a null ES for each set. The LES is defined as the core grouping of genes contributing to the enrichment score; this generally represents approximately 30–50% of genes in an enriched category [21].

### Validation of expression patterns

Three ESTs deemed to be differentially expressed, using the LIMMA package of Bioconductor, plus one on the threshold of significance were chosen for validation of array predictions by real time PCR analyses. The ESTs and primers used are outlined in Table S1. The Primer Express package (Applied Biosystems) was used to design primers to the four ESTs and the 18S ribosomal RNA control. A dissociation plot was performed for each primer to determine specificity. Comparable amplification was confirmed and assays performed in triplicate, on an ABI 7300 PRISM instrument using SYBR green dye, according to the manufacturer's instructions. All data were normalized to the lowest level of expression as determined by real time PCR.

## Results

### Single gene analysis reveals few significant differences between normal and irradiated parasites

The LIMMA package analysis of changes in single genes across the three time points highlights only seven significant differences between irradiated and normal parasites (Table 1). In all cases genes reaching our stringent statistical cut-off are conspicuously down-regulated in the irradiated parasite. Two differentially regulated transcripts encode proteins destined for the plasma membrane. One of these is the previously characterised Sm25 (also known as Gp18–22) and the other codes for a hypothetical protein. A third transcript encoding Tetraspanin D (Sm-TSP-2) , known to be present at the tegumental surface [22], is down-regulated at all three time points. Of the remaining genes revealed, JF-2 codes for a membrane-associated cytoskeletal component thought to link actin filaments to the plasma membrane, cdc2 is a key control enzyme of the cell cycle and two code for hypothetical proteins (Table 1).

**Table 1:** Individual genes down-regulated in the irradiated parasite.

Stage	Contig^1^	Log odds^2^	Predicted subcellular location	Annotation	Accession#^3^
4	Sm12366	6.2	lysosome	Tetraspanin D	Q8ITD7
4	Sm04760	3.958	plasma membrane	Gp18-22	Q7JPY1
7	Sm12366	6.087	lysosome	Tetraspanin D	Q8ITD7
10	Sm12366	18.546	lysosome	Tetraspanin D	Q8ITD7
10	Sm12949	8.132	No Prediction	Hypothetical	N/A
10	Sm05076	3.433	nucleus	cdc2	O17507
10	Sm06902	3.243	cytoplasm	JF-2	Q26520
10	Sm03463	3.149	plasma membrane	Hypothetical	N/A
10	Sm29577	3.09	No Prediction	Hypothetical	N/A
all	Sm12366	6.2	lysosome	Tetraspanin D	Q8ITD7
all	Sm04760	3.958	plasma membrane	Gp18-22	Q7JPY1

1 Sm numbers are contigs and singlet sequences available at http://www.genedb.org/genedb/smansoni/

2 ln probability that a gene is differentially expressed divided by the ln probability it is not. (Approximately P≤0.0001)

3 BLAST hit from the UniProt database on which putative function was assigned.

### Real time PCR confirms single gene analysis

The level of expression of the four selected ESTs was determined using real time RT-PCR and compared with that estimated from the array hybridisations (Figure S1). Plotting the data as a histogram highlights the broad level of agreement between the two techniques, and shows that in contrast to previous work [14], differences in sensitivity are not as pronounced. This is likely due to the smaller variations in expression measured. A scatter plot of the same data (data not shown) demonstrates that the two methods exhibited high concordance, with a correlation coefficient R = 0.80.

### GSEA highlights extensive and subtle differences in gene expression

**Figure 1:**
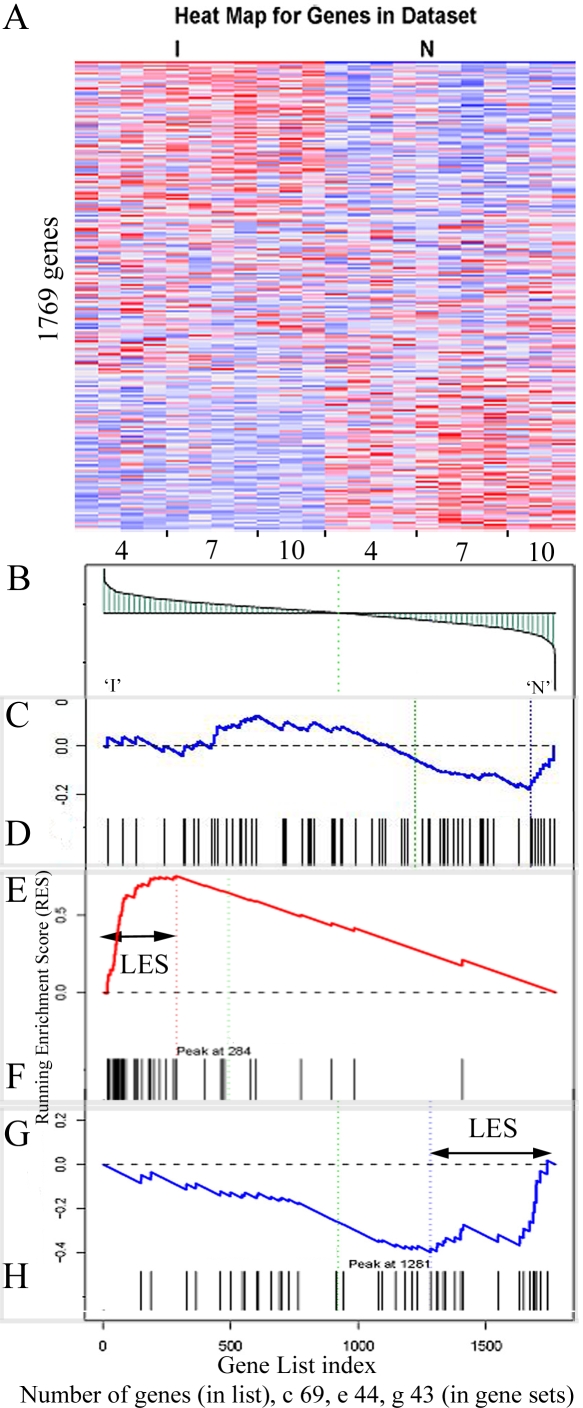
Illustrative examples of the Gene Sets Enrichment Analysis. A. heat map showing genes ranked according to differences in expression profile that correlate with either the irradiated (I) or normal (N) phenotype; x-axis labels indicate sampling times in days. B. the ranked gene list as a graphical correlation of expression with the two phenotypes; the y-axis indicates the deviation from the mean expression level and the green line denotes the zero crossing point. Graphical examples of the running sum statistic for three gene sets are shown the black bars indicate the position of each transcript in a given set relative to the ranked gene list: C. and D. no enrichment, 69 gene set ‘protein kinase activity’, day seven; E. and F. irradiated parasite enrichment, 44 gene set ‘structural constituent of ribosome’, day ten; G. and H. normal parasite enrichment, 43 gene set ‘receptor activity’, all stages. Leading Edge Subsets are defined by the respective maximum or minimum deviation from zero, indicated by double headed arrows where appropriate.

The expression of genes, grouped by biological function or subcellular location, correlating with a specific phenotype, was assessed using the GSEA package. A heat map recording the differential expression of the 1769 unique features on the array is presented in Figure 1A. A symmetrical distribution of expression profiles, can be discerned whereby two thirds of the genes are visibly associated with a phenotype. Approximately one third are up-regulated in the irradiated parasite (the red-dominated top left corner) and a different third in the normal parasite (the red-dominated bottom right corner); the remaining central third display no obvious pattern. A list of genes ranked by the intensity of their expression was derived from the heat map (Figure 1B) and used to produce a graphical plot of the running sum statistic (Figure 1C, E and G). This running sum statistic increases every time a member of a given gene set (i.e. a GO category) is encountered in the ranked gene list and decreases when it is not encountered. Where no correlation occurs between a gene set and the N or I phenotype the genes appear randomly in the ranked list producing a plot of the running sum statistic that fluctuates either side of zero (e.g. Figure 1C). The running sum for expression of the gene sets that correlate with the irradiated phenotype (e.g. Figure 1E) is skewed to the left by the abundance of numerous members in that region of the ranked gene list (Figure 1F). Conversely, correlation with the normal phenotype is skewed to the right (e.g. Figure 1G and H). The leading edge subset (LES) represents the core of genes most strongly associated with the N or I phenotype (Figure 1E and G).

### Protein catabolism, retrotransposon activity and G-protein signalling are differentially enriched at day four

The most prominent enriched gene sets at day four include ‘protein modification’ in the irradiated parasite and ‘RNA-directed DNA polymerase activity’ (root GO term is ‘Molecular Function’, which is identical to the ‘Biological process’ category ‘RNA-dependent DNA replication‘) in the normal parasites (Table 2). The former category is noteworthy for containing the E1-3 ubiquitinating enzymes in its LES (Table S2) while the latter appears to consist primarily of retrotransposon transcripts (at least 17 members, data not shown). The ‘GTP binding’ LES associated with the irradiated phenotype contains no fewer than 17 ras/rab/rac small G-protein homologues, together with a stimulatory and an inhibitory heterotrimeric G-protein alpha subunit (Table S2). A fifth ‘calcium ion binding’ category, also associated with the irradiated phenotype, possesses numerous transcripts encoding proteins of disparate motor or structural function including EF hand-containing proteins such as Sm22.6, myosin, at least two annexins and severin (Table S2).

**Table 2:** GSEA categories associated with a given phenotype at each time point.

Category	Description	Phenotype, False Discovery Rate*			
		Day 4	Day 7	Day 10	Day 4–10
**Biological Process**					
GO:0003735	Structural constituent of ribosome		I, 0.21	I, 0.07	I, 0.06
GO:0005975	Carbohydrate metabolism				I, 0.08
GO:0006118	Electron transport		I, 0.07		
GO:0006278	RNA-dependent DNA replication	N, 0.13		N, 0.23	
GO:0006412	Protein biosynthesis		I, 0.20		I, 0.15
GO:0006457	Protein folding		I, 0.21		I, 0.25
GO:0006464	Protein modification	I, 0.20			
GO:0006468	Protein amino acid phosphorylation			N, 0.16	
GO:0006508	Proteolysis		I, 0.20		
GO:0006810	Transport			N, 0.13	
GO:0006811	Ion transport			N, 0.16	
GO:0006812	Cation transport			N, 0.24	
GO:0007155	Cell adhesion				N, 0.19
GO:0007186	G-protein coupled receptor protein signaling pathway			N, 0.20	
GO:0007242	Intracellular signaling cascade			N, 0.24	N, 0.17
GO:0007264	Small GTPase mediated signal transduction		I, 0.19		I, 0.05
GO:0008152	Metabolism		I, 0.23		
GO:0015031	Protein transport			N, 0.13	
**Cellular Component**					
GO:0005737	Cytoplasm			N, 0.18	
GO:0005739	Mitochondrion		I, 0.21		
GO:0005794	Golgi apparatus			N, 0.23	
GO:0005829	Cytosol				I, 0.24
GO:0005840	Ribosome		I, 0.23	I, 0.07	I, 0.05
GO:0005856	Cytoskeleton			N, 0.13	N, 0.23
GO:0006355	Regulation of transcription, DNA-dependent			N, 0.14	
GO:0016020	Membrane			N, 0.19	
GO:0016021	Integral to membrane			N, 0.13	
**Molecular Function**					
GO:0000166	Nucleotide binding			N, 0.13	
GO:0000287	Magnesium ion binding			N, 0.14	
GO:0003677	DNA binding			N, 0.18	
GO:0003723	RNA binding			N, 0.18	
GO:0003964	RNA-directed DNA polymerase activity	N, 0.13		N, 0.23	
GO:0004386	Helicase activity			N, 0.13	
GO:0004672	Protein kinase activity			N, 0.13	
GO:0004674	Protein serine/threonine kinase activity			N, 0.23	N, 0.24
GO:0004871	Signal transducer activity				N, 0.19
GO:0004872	Receptor activity			N, 0.15	N, 0.15
GO:0005198	Structural molecule activity			N, 0.19	
GO:0005216	Ion channel activity			N, 0.14	N, 0.23
GO:0005489	Electron transporter activity		I, 0.18		I, 0.09
GO:0005509	Calcium ion binding	I, 0.23			
GO:0005515	Protein binding			N, 0.14	N, 0.19
GO:0005525	GTP binding	I, 0.13			I, 0.01
GO:0008234	Cysteine-type peptidase activity		I, 0.21		I, 0.06
GO:0016301	Kinase activity				N, 0.23
GO:0016491	Oxidoreductase activity		I, 0.13		
GO:0016740	Transferase activity			N, 0.17	N, 0.25
GO:0016853	Isomerase activity		I, 0.09		I, 0.04
**PA**					
n/a	ER			N, 0.14	N, 0.23
n/a	Extracellular		I, 0.22		
n/a	Mitochondrion		I, 0.23		
n/a	Plasmamembrane			N, 0.25	
**Miscellaneous**					
GO:0005554	Molecular function unknown			N, 0.16	

Columns indicate which gene set correlates with a normal (N) or irradiated (I) phenotype; the FDR associated with each correlation follows after a comma.

*: False Discovery Rate (FDR) denotes the probability that the association is spurious.

### Aerobic and protein metabolism are enriched in the day seven irradiated parasite

At day seven, only the irradiated phenotype shows gene set enrichment (Table 2). Gene categories associated with ‘metabolism’, ‘mitochondrion’ and ‘electron transport’ are overrepresented and an analysis of LES overlap reveals commonalities between the three subsets of enriched genes. The genes shared are specifically involved in the respiratory electron transport chain. A number of cytochrome subunits, NADH metabolising and antioxidant thioredoxin enzymes all contribute to the enrichment score of the three gene sets (Table S3). Protein synthesis and degradation also appear to be prominent processes in the day seven irradiated parasite. The categories ‘protein biosynthesis’, ‘protein folding’, ‘proteolysis’, ‘ribosome’, ‘structural constituent of ribosome’, ‘cysteine-type peptidase activity’ and ‘isomerase activity’ encompassing genes encoding translation initiation factors, isomerases and chaperones all correlate with the irradiated parasite phenotype (Table 2). Intriguingly, transcripts encoding extracellular proteins also appear to be enriched, although the heterogeneous nature of this LES makes it difficult to discern a biological pattern (Table S3). Nevertheless, the presence of the antigen 5 transcript, protease inhibitors and a lipoprotein receptor is noteworthy.

### The irradiated parasite transcriptome diverges further from the norm by day ten

The transcriptional divergence of the irradiated and normal parasites is even more apparent by day ten; 32 of the 89 gene sets submitted to GSEA show enrichment correlating with one or other phenotype. For the irradiated parasite the enrichment of ‘ribosome’ components persists into day ten and ‘RNA-directed DNA synthesis’ is again comparatively depressed with respect to the normal parasite. Indeed, at this stage the irradiated parasite differs from the normal parasite in many biological systems (Table 2). Categories for ‘transcription regulation’, ‘RNA binding’ and ‘helicase activity’ are under-represented in the irradiated parasite and there is also a relative shortfall in ‘intracellular kinase signalling’ and ‘structural proteins’, specifically cytoskeletal transcripts. The comparative paucity of receptor-encoding transcripts is particularly striking in the irradiated parasite, as is the general dearth of transcripts from the gene sets ‘endoplasmic reticulum’, through the ‘golgi’ to the ‘plasma membrane’. An analysis of the LES of the ‘receptor activity’ category reveals an overlap with other gene sets diminished in their own right, including ‘ion channel activity’ and ‘G-protein coupled receptor signalling’. The functional overlap reveals that a significant proportion of these transcripts are neuroreceptors or channels, including acetylcholine, purinergic, nicotinic, glutamate and aspartate receptors plus voltage and ligand-gated ion channels (Table S4).

### Pooled data highlights the core deficits of the irradiated parasite

Examining the pooled data for differential enrichment of categories, in normal versus irradiated, emphasises the apparent importance of protein synthesis and degradation in the irradiated parasite. Categories associated with protein metabolism, including ‘protein folding’ are prominent as is the ‘cysteine-type peptidase activity’ GO set, containing a number of cathepsins, and a ‘cytosol’ set that contains proteosome activators and some 20S proteosome components in its LES (Table S5). While Golgi-related transcripts do not meet the FDR cut-off, the overlap between the ‘golgi’, ‘GTP binding’ and ‘small GTPase mediated signal transduction’ LES together with deficiencies in the ‘ER’ category is noteworthy (Table S5). Although the comparative paucity of ‘receptor activity’ transcripts in the irradiated parasite is not obvious at days four and seven, the receptor activity is depressed at all time points when irradiated versus normal parasites were compared (Table 2). Analysis of the category ‘ion channel activity’ comprising transcripts encoding receptors associated with ion flux across membranes is also diminished in the irradiated parasite. Signalling cascades, particularly ‘kinase activity’ (Table 2) are also less abundant in the irradiated parasite. The kinases may well interact with the ‘cell adhesion’ and ‘cytoskeleton’ categories contributing to the observed differences (Table 2) but overlap analysis does not indicate shared genes in their respective LES.

## Discussion

Lung stage schistosomula of *S. mansoni* are a validated target of protective immunity induced in the murine host by exposure to RA cercariae. However, attempts to identify the antigens responsible, a key step in the development of a recombinant vaccine, have met with limited success [1],[8]. Microarrays offer a route to antigen identification by pinpointing subtle differences in gene expression between irradiated and normal worms, irrespective of transcript abundance. Characterising the underlying transcriptional differences should highlight changes at the parasite-host interface that explain why irradiated larvae can elicit protective immunity when normal larvae do not. In addition, by shifting the focus away from antibody-based technologies, microarrays may identify genes encoding non-immunogenic proteins that are nevertheless fundamental to parasite migration and development.

Analysis of the normal and irradiated parasite transcriptomes at day four, seven and ten revealed only seven genes that showed significant differences in expression. All were down-regulated as a result of radiation. Proteome Analyst predicted two as plasma membrane proteins (Sm25; hypothetical protein). A third, a tetraspanin (Sm-TSP-2, CD63-like, tetraspanin D), mispredicted as lysosomal, is known to be exported to the tegument surface plasmamembrane [22], as is Sm25 [23],[24]. Biotinylation studies on adult worms indicated that tetraspanin D may play a role in maintaining tegumental membrane structure and organisation and could be accessible to the immune system [22]. Indeed, this particular tetraspanin, identified using a signal sequence trap [25], elicited protective immunity when the major extracellular loop was used to vaccinate mice [26]. Conversely Sm25, or its decorating glycans, may actually protect the parasite by subverting the host immune response as, despite eliciting high antibody titres, the recombinant protein does not protect vaccinated animals [27]. On the basis of membrane association and immunofluorescence studies it has been suggested that the actin binding protein JF-2 may be available at the tegument surface [28]. However, a sizeable proportion of patients infected with *S. japonicum* possess antibodies to JF-2 [28], yet continual chemotherapy is still required to limit the impact of reinfection [29]. This observation argues that JF-2 normally confers little or no resistance and may simply be another cytoplasmic protein albeit one associated with plasma membranes [30]. Cdc2, the final protein with an ascribed function, is a crucial cell cycle control enzyme. While the down-regulation of a single gene should not be over interpreted, suppressed levels of the cdc2 protein may reflect the inability to re-enter the cell cycle [31]. Migrating schistosomes are in a semi-quiescent metabolic state (Lawson and Wilson, 1980) with no cell division taking place [32]. However, they are primed to enter cell cycle upon reaching the portal vein and beginning to blood feed [33]. Thus, down-regulation of cdc2 may be part of the explanation why irradiated parasites never mature.

It is clear from numerous studies on the RA vaccine (reviewed by Coulson 1997) that attenuated parasites must persist in the host for 1–2 weeks to elicit effective protection. Furthermore they must also migrate beyond the skin to its draining lymph nodes, and to the lungs. As anticipated, large transcriptional changes were not evident four days or more after the radiation insult, since the acute stress response has long subsided by that time [34]. Therefore, the ability to detect small coordinated changes, using the GSEA package developed by Subramanian *et al.* was particularly important as a means of dissecting out the longer-term effects of radiation exposure. Schistosomula undergo marked phenotypic changes while resident in the skin soon after penetration, which include remodelling of the tegument surface and ablation of penetration glands [35]. Subsequently, mid-body spines are lost and the larval body elongates to facilitate intravascular migration beyond the lungs [36]. At day four, approximating to the skin stage, it was difficult to detect meaningful differences in transcript abundance, suggesting that the delayed effects of radiation were very subtle. However, RNA-directed DNA synthesis, indicated by retrotransposon transcription, was more prominent in the normal parasite. Why this should be depressed in the irradiated parasite is unclear but could reflect long term suppression by DNA repair mechanisms [37]. The ‘protein modification’ category provided an early indication of enhanced protein metabolism in the irradiated parasite. By day seven the protein metabolism categories specified by GSEA revealed a more pronounced effect but this distinction was diminished by day ten. Despite the lack of obvious morphological differences between early normal and irradiated parasites (Mastin *et al.*, 1983) our data are consistent with the observations of Wales *et al* (1992) that protein synthesis is temporarily inhibited by irradiation. It seems likely that body remodelling has been delayed so the enhanced protein metabolism may reflect a catch-up process relative to the normal parasite. In a similar vein the switch to anaerobic respiration [38] may be retarded as evidenced by the enrichment of energy metabolism categories at day seven, in the irradiated parasite.

By day ten the divergence between normal and irradiated parasites was greatest, with the majority of highlighted gene sets down-regulated in the latter. We consider that these represent biological processes damaged beyond recovery by the now-distant radiation event. The decreased prominence of categories involving intracellular signalling (e.g. ‘G-protein coupled receptor signalling pathway’) may indicate a reduced ability to respond to external developmental cues; the down-regulation of cdc2, already noted, should be viewed in this context. In addition, deficiencies in structural categories such as ‘cytoskeleton’ may further impede the irradiated parasite's capacity for locomotion. This apparent inability to detect and respond appropriately to the surroundings is further reinforced by the comparative paucity of receptor transcripts, especially those encoding components of neurotransmitter pathways. All these categories identified by GSEA accord with the visible phenotype revealed by SEM studies [3]. Although the irradiated parasite is in most respects morphologically similar to the normal parasite, elongating and losing mid-body spines [39], it nevertheless displays abnormal constrictions of circular muscle fibres in the body wall, resulting in uncoordinated movement [3]. It is this compromised locomotion that leads to the persistence of irradiated parasites in the host lymph nodes and lungs for five weeks or more [4],[39]. In the lymph nodes the parasites drive lymphocyte proliferation [40] and in the lungs they act as a long-term stimulus to recruit lymphocytes that arm that organ against challenge parasites [41].

The reason that RA parasites in general elicit protective immunity when normal parasites do not has long been the subject of speculation and investigation [42]. Our study strongly indicates that the up-regulation of specific gene products to provide elevated immune stimulation is not the key. Indeed an expressed fragment of the tetraspanin gene that we detected as down-regulated by single gene analysis, was recently shown to have protective potential in the mouse [26]. This underlines our thesis that even if gene expression is reduced, the extended stay of attenuated parasites in the skin draining lymph nodes may still result in enhanced immune priming against exposed antigens. Equally once the host has been primed by the vaccine, antibody or cell mediated effector responses could act early upon the incoming parasite, after cercaria-schistosomulum transformation has been completed; from previous microarray experiments we already know that tetraspanin D is strongly expressed in the two day old schistosomulum [14].

We cannot rule out that parasites *in vivo* respond differently to some host factor, not present *in vitro,* by up-regulating specific genes as suggested by *ex vivo* experiments [43]. However, the subtle nature of differences between normal and irradiated parasites leads us to believe that changes in protein expression are poor indicators of potential antigenicity; it is likely that the accessibility rather than abundance of an antigen is the important factor. In this context, retarded development increasing the duration of immune stimulation appears to be the salient feature. In the long term, the radiation insult compromises the transcription of schistosome genes involved in neuromuscular activity and ultimately cell cycle progression. In this respect schistosomes are particularly suited to deliver a prolonged stimulus as they undertake a protracted migration from skin to portal system, lasting 7–21 days after penetration (i.e. irradiation). Only when blood feeding and cell division begin in the liver [44] will DNA strand breaks prove lethal. This priming by larvae is quite distinct in both location and antigen load from the continuous priming over months to years provided by adult worms and their eggs. Furthermore, recent studies in the baboon model have shown that protective responses elicited by the irradiated vaccine are dissociated from both responses to chemotherapy and an ongoing chronic infection [45].

Exposure to irradiated metazoan and protozoan parasites has been widely used to study protective immunity, as the basis for vaccine development, but we believe this is the first attempt to interrogate the transcriptome of such a parasite. In addition to schistosomes, protective immunity is induced by radiation-attenuation of the nematodes *Dictyocaulus* and *Ancylostoma spp.* and the protozoa, *Plasmodium, Eimeria* and *Theileria spp.* [46]–[50]. Given the efficacy of radiation-attenuated parasites as vaccines, the findings of this study should provide pointers to the phenotypic changes that account for the success of these other parasites as inducers of protective responses.

## Supporting Information

Table S1 Primers used in real time PCR.
(0.03 MB DOC)

Table S2 Genes in the leading edge subset of the 'protein modification', 'GTP binding' and 'calcium ion binding' categories.
(0.08 MB DOC)

Table S3 Genes in the leading edge subset of the 'metabolism', 'mitochondrion', 'electron transport' and 'extracellular' categories. Cytochrome c oxidase appears to have been mis-assigned to this set by Proteome Analyst.
(0.10 MB DOC)

Table S4 Genes with a putative neurological function present in the leading edge subset of the 'receptor activity' category.
(0.03 MB DOC)

Table S5 Leading edge subset of the 'cytosol', 'Golgi', 'GTP binding' and 'small GTPase mediated signal transduction' GO and proteome analyst categories
(0.09 MB DOC)

Figure S1 To validate array data qPCR was performed on four representative genes for comparison. The bar chart illustrates the fold differences determined by the two methods. Only qPCR produces means +/− SEM.
(3.00 MB TIF)
