# Supplementary material for: Altered Patterns of Gene Expression Underlying the Enhanced Immunogenicity of Radiation-Attenuated Schistosomes
Source: PLoS Negl Trop Dis. 2008 May 21;2(5):e240. doi: 10.1371/journal.pntd.0000240 (PMC2375114; doi:10.1371/journal.pntd.0000240)
Supplement: Table S1 — Primers used in real time PCR. (0.03 MB DOC) [file pntd.0000240.s001.doc]

Table S1: Primers used in real time PCR.

| Gene product | Forward primer | Reverse primer |
| --- | --- | --- |
| 18S | TCGGCGACGGATCTTTCA | CCGGAATCGAACCCTGATTC |
| Sm12949 | TCACAGCTCGTGAAACTCAACAA | ACAATGAATATGTGACTCGATACT |
| Sm05076 | GGCACGAGGGCGACAAG | CTGCACCGAATTGGTAAGAAAAC |
| Sm06588 | TTATGACAGATGAAGGCTTCCAAA | GGTGCAGAAGCAGCTGAATG |
| Sm12366 | TTCGCTCCAGTGATTTACAACAG | CATATGTGGTCTCGGGCTGAT |
