# Supplementary material for: Altered Patterns of Gene Expression Underlying the Enhanced Immunogenicity of Radiation-Attenuated Schistosomes
Source: PLoS Negl Trop Dis. 2008 May 21;2(5):e240. doi: 10.1371/journal.pntd.0000240 (PMC2375114; doi:10.1371/journal.pntd.0000240)
Supplement: Table S2 — Genes in the leading edge subset of the 'protein modification', 'GTP binding' and 'calcium ion binding' categories. (0.08 MB DOC) [file pntd.0000240.s002.doc]

Table S2: Genes in the leading edge subset of the ‘protein modification’, ‘GTP binding’ and ‘calcium ion binding’ categories.

| Gene | Putative product | Uniprot Accession |
| --- | --- | --- |
|  | **‘Protein modification’** |  |
| Sm03337 | Lysosomal alpha-mannosidase precursor | O00754 |
| Sm01588 | E3 ubiquitin protein ligase homologue 1 | Q7Z6Z7 |
| Sm12980 | E2 ubiquitin-conjugating enzyme | Q86E16 |
| Sm09824 | E3 ubiquitin protein ligase homologue 2 | Q7TMY8 |
| Sm03242 | E1 ubiquitin-activating enzyme | Q7ZVX6 |
|  | **‘GTP binding’** |  |
| Sm11292 | Dynamin-1 | P21575 |
| Sm03786 | Ras-related protein Rab-11A (Rab-11) | P62493 |
| Sm12227 | Ras-related protein Rab-2A | P53994 |
| Sm13242 | Elongation factor G (EF-G) | Q8DI43 |
| Sm02151 | Putative SRP | Q7PSJ1 |
| Sm11682 | Rab family protein 1 | Q86ET1 |
| Sm29703 | Probable Ras-related protein Rab-4A | Q9GP33 |
| Sm03589 | ADP-ribosylation factor, arf, putative | Q86E39 |
| Sm11213 | Ras-related protein Rab-14 | Q86EY7 |
| Sm01205 | Ras-related protein Rab-6 homolog F59B2.7 | P34213 |
| Sm11361 | Guanine nucleotide-binding protein G(q), alpha subunit | P38411 |
| Sm04990 | ADP-ribosylation factor | Q86F33 |
| Sm04550 | Ras-related protein Rap-1b (GTP-binding protein smg p21B) | Q99JI6 |
| Sm01350 | Rab4A-like protein | O44213 |
| Sm06260 | Guanine nucleotide-binding protein G(s) subunit alpha | P30669 |
| Sm00509 | Rab-related GTP-binding protein | Q26554 |
| Sm01081 | Initiation factor 2 subunit | Q86E66 |
| Sm13217 | Ras related small G protein RAL-A (RALA protein) | Q9I8H8 |
| Sm01001 | Rac GTPase | Q8I898 |
| Sm00524 | Ras-related protein Rab-2 | Q05975 |
| Sm03509 | Ras-related protein M-Ras (Ras-related protein R-Ras3) | Q86F60 |
| Sm00043 | Hypothetical protein (Fragment) | Q6DKL2 |
| Sm05095 | Ras-related protein Rab | Q86ER2 |
| Sm06722 | Rac GTPase | Q8I898 |
| Sm10788 | Signal recognition particle receptor | Q7PSJ1 |
|  | **‘Calcium ion binding’** |  |
| Sm04483 | EF-hand containing | Q86FI4 |
| Sm06723 | Myosin light chain | Q9Y1U7 |
| Sm12876 | Tegument antigen SMA22.6 | P14202 |
| Sm03739 | egf-like domain protein | Q23587 |
| Sm12852 | Annexin | Q9XY89 |
| Sm09495 | EF-hand containing | O44117 |
| Sm01247 | Sorcin | Q94743 |
| Sm07755 | Calmodulin (CaM) | O16305 |
| Sm03497 | Hypothetical protein F10C2.5 | P90830 |
| Sm12742 | Severin | Q24800 |
| Sm10308 | FLJ13397 protein | Q6IA40 |
| Sm12418 | 20 kDa calcium-binding protein (Antigen SM20) | P15845 |
| Sm12950 | Sorcin | Q94743 |
| Sm10663 | Leucine zipper-EF-hand containing transmembrane protein 1 | O95202 |
| Sm01066 | Myosin regulatory light chain, putative | Q86EK2 |
| Sm12814 | Leucine zipper-EF-hand containing transmembrane protein 1 | Q7Q0I8 |
| Sm05256 | Calcineurin B | Q86H16 |
| Sm10144 | Neurogenic locus notch homolog protein 3 precursor (Notch 3) | Q9R172 |
| Sm01497 | IRRADIATED VACCINE antigen 1 precursor | Q04702 |
| Sm03694 | Hypothetical | Q60902 |
| Sm29991 | Ankyrin repeat domain protein 5 | Q9NU02 |
| Sm01709 | Annexin | Q9XY89 |
| Sm12997 | Severin | Q24800 |
| Sm04123 | Severin | Q24800 |
| Sm05329 | Hypothetical | Q86EE1 |
| Sm13117 | Annexin | Q9XY89 |
| Sm04737 | EF-hand containing | Q86EE1 |
