# Supplementary material for: Altered Patterns of Gene Expression Underlying the Enhanced Immunogenicity of Radiation-Attenuated Schistosomes
Source: PLoS Negl Trop Dis. 2008 May 21;2(5):e240. doi: 10.1371/journal.pntd.0000240 (PMC2375114; doi:10.1371/journal.pntd.0000240)
Supplement: Table S3 — Genes in the leading edge subset of the 'metabolism', 'mitochondrion', 'electron transport' and 'extracellular' categories. Cytochrome c oxidase appears to have been mis-assigned to this set by Proteome Analyst. (0.10 MB DOC) [file pntd.0000240.s003.doc]

Table S3: Genes in the leading edge subset of the ‘metabolism’, ‘mitochondrion’, ‘electron transport’ and ‘extracellular’ categories. Cytochrome c oxidase appears to have been mis-assigned to this set by Proteome Analyst.

| Gene | Putative product | Uniprot Accession |
| --- | --- | --- |
|  | **‘Metabolism’** |  |
| Sm13268 | Solute carrier family 27 (Fatty acid transporter), member 4 | Q6P1M0 |
| Sm00999 | Triosephosphate isomerase (EC 5.3.1.1) (TIM) | P48501 |
| Sm04345 | Phosphoserine phosphohydrolase-like protein trans-spliced | Q26545 |
| Sm29118 | Hypothetical | Q18143 |
| Sm03875 | Hypothetical | Q7P407 |
| Sm11799 | Ca-ATPase-like protein SMA3 | Q967W1 |
| Sm11467 | Putative undecaprenyl diphosphate synthase | Q86E48 |
| Sm01456 | Putative Acyl transferase | Q7PNS5 |
| Sm01315 | aldehyde dehydrogenase, putative | Q7Q165 |
| Sm04097 | Hypothetical | O61143 |
| Sm13015 | Hypothetical | P44898 |
| Sm07278 | aconitase, mitochondrial, putative | Q7Q3F6 |
| Sm03441 | Phosphoglycerate mutase | Q8WT66 |
| Sm01697 | Carbonyl reductase-like protein trans-spliced | Q26546 |
| Sm12954 | Hypothetical | O17412 |
| Sm08594 | Hypothetical protein F09C3.2 | O17773 |
| Sm11819 | Glutamine amidotransferase, class-II | Q7PLC6 |
| Sm08982 | short-chain dehydrogenase, putative | Q7Q686 |
|  | **‘mitochondrion’** |  |
| Sm01482 | Cytochrome oxidase c subunit | Q6XHZ3 |
| Sm12762 | Mitochondrial 28S ribosomal protein S28 (S28mt) (MRP-S28) | Q9CY16 |
| Sm11953 | Ubiquinol-cytochrome C reductase complex 14 kDa protein | O01374 |
| Sm11752 | Putative NADH-ubiquinone oxidoreductase 23 kDa subunit, mitochondrial precursor | Q8K3J1 |
| Sm04280 | Mitochondrial 28S ribosomal protein S2 (MRP-S2) | Q8BQ99 |
| Sm06152 | Putative Pyruvate dehydrogenase E1 component beta subunit, mitochondrial | Q38799 |
| Sm00929 | NADH dehydrogenase subunit 5 | Q9B8X5 |
| Sm13311 | Cytochrome c oxidase subunit 1 (Fragment) | Q70W85 |
| Sm01048 | Cytochrome c oxidase subunit 1 | Q9B8X8 |
| Sm00455 | Hypothetical protein W10D9.5 | O17287 |
| Sm00668 | Putative peptidase | Q8BHT9 |
| Sm09098 | NADH-ubiquinone oxidoreductase B18 subunit | Q7QJL9 |
| Sm08957 | Putative ATP-binding cassette, sub-family B, member 7, mitochondrial precursor | O75027 |
| Sm00771 | Cytochrome c oxidase subunit 3 | Q9B8X4 |
| Sm03206 | Putative DnaJ homolog subfamily A member 3, mitochondrial precursor | Q96EY1 |
| Sm00970 | Cytochrome c oxidase subunit 2 | Q9MD49 |
| Sm00726 | Hypothetical | Q86DY6 |
| Sm07278 | Aconitase 2, mitochondrial | Q7Q3F6 |
| Sm04456 | Hypothetical | Q92667 |
| Sm12324 | Cytochrome b | Q9B8X3 |
| Sm07721 | Hypothetical | Q8HB19 |
| Sm02331 | Hypothetical | O45503 |
| Sm08286 | Putative Mitochondrial deoxynucleotide carrier | Q9HC21 |
|  | **‘electron transport’** |  |
| Sm01482 | Cytochrome oxidase c subunit | Q6XHZ3 |
| Sm12037 | Fad oxidoreductase, putative | Q89GA3 |
| Sm08285 | Protein disulfide isomerase (Fragment) | O96460 |
| Sm11953 | Ubiquinol-cytochrome C reductase complex 14 kDa protein (EC 1.10.2.2) | O01374 |
| Sm12927 | Mitosis protein DIM1 | Q7Q496 |
| Sm12512 | Thioredoxin | Q7PEL6 |
| Sm11752 | NADH-ubiquinone oxidoreductase 1, chain, putative | Q8K3J1 |
| Sm11767 | Thioredoxin | Q8T9N5 |
| Sm12752 | Cytochrome b5 reductase 1 | Q86FI5 |
| Sm00980 | Protein disulfide isomerase homologue precursor | Q26593 |
| Sm03498 | Lysine-specific histone demethylase 1, putative | Q6ZQ88 |
| Sm00446 | Succinate dehydrogenase complex, subunit A, flavoprotein (Fp) | Q7ZVF3 |
| Sm03361 | SCO1 protein homolog, mitochondrial precursor | Q7PPN7 |
| Sm01048 | Cytochrome c oxidase subunit 1 | Q9B8X8 |
| Sm12956 | Hypothetical | Q95AS9 |
| Sm00414 | Pyridine nucleotide-disulphide oxidoreductase | Q7PYX7 |
| Sm04097 | Hypothetical | O61143 |
| Sm09098 | NADH-ubiquinone oxidoreductase B18 subunit | Q7QJL9 |
| Sm00771 | Cytochrome c oxidase subunit 3 | Q9B8X4 |
| Sm00970 | Cytochrome c oxidase subunit 2 | Q9MD49 |
| Sm09091 | Cytochrome c1 | Q6VBC0 |
| Sm12324 | Cytochrome b | Q9B8X3 |
|  | **‘Extracellular’** |  |
| Sm00285 | Gynecophoral canal protein | Q26557 |
| Sm05361 | Cystatin precursor | P35481 |
| Sm01048 | Cytochrome c oxidase subunit | Q9B8X8 |
| Sm12764 | Antigen 5 homologue | Q86FG7 |
| Sm07289 | Immunoglobulin-like hypothetical protein 1 | Q86FD7 |
| Sm05195 | Hypothetical protein | Q8IDA6 |
| Sm13109 | Serpin | Q26606 |
| Sm01352 | Immunoglobulin-like hypothetical protein 2 | Q86FD7 |
| Sm01246 | WW domain motif | O16786 |
| Sm07396 | Lipoprotein-receptor related protein | O16148 |
| Sm13225 | Proline-rich hypothetical protein | Q6ZMN3 |
