# Supplementary material for: Altered Patterns of Gene Expression Underlying the Enhanced Immunogenicity of Radiation-Attenuated Schistosomes
Source: PLoS Negl Trop Dis. 2008 May 21;2(5):e240. doi: 10.1371/journal.pntd.0000240 (PMC2375114; doi:10.1371/journal.pntd.0000240)
Supplement: Table S4 — Genes with a putative neurological function present in the leading edge subset of the 'receptor activity' category. (0.03 MB DOC) [file pntd.0000240.s004.doc]

Table S4 Genes with a putative neurological function present in the leading edge subset of the ‘receptor activity’ category.

| Gene | Putative product | Uniprot Accession |
| --- | --- | --- |
| Sm30055 | Acetylcholine receptor protein, gamma chain precursor | P04760 |
| Sm29692 | Putative G-protein coupled receptor | Q8MTW6 |
| Sm04043 | Hypothetical P2X purinoceptor 4 | Q99571 |
| Sm01261 | Nicotinic acetylcholine receptor precursor 1 | Q6TMX8 |
| Sm28782 | Nicotinic acetylcholine receptor precursor 2 | Q6TMX8 |
| Sm01385 | Hypothetical N-methyl D-aspartate receptor | Q8CG67 |
| Sm01384 | Hypothetical Odorant receptor 13.3 | Q9I8Z3 |
| Sm28996 | ATP-gated ion channel subunit P2X4 | Q9DDP0 |
| Sm12784 | Lipocalin homologue | Q9D1E5 |
| Sm11995 | Hypothetical acetylcholine receptor | P54245 |
| Sm11063 | Hypothetical glutamate receptor | Q7QCT5 |
| Sm01116 | Neurotransmitter-gated ion-channel ligand-binding | Q9NZR2 |
