# Supplementary material for: Altered Patterns of Gene Expression Underlying the Enhanced Immunogenicity of Radiation-Attenuated Schistosomes
Source: PLoS Negl Trop Dis. 2008 May 21;2(5):e240. doi: 10.1371/journal.pntd.0000240 (PMC2375114; doi:10.1371/journal.pntd.0000240)
Supplement: Table S5 — Leading edge subset of the 'cytosol', 'Golgi', 'GTP binding' and 'small GTPase mediated signal transduction' GO and proteome analyst categories (0.09 MB DOC) [file pntd.0000240.s005.doc]

Table S5: Leading edge subset of the ‘cytosol’, ‘Golgi’, ‘GTP binding’ and ‘small GTPase mediated signal transduction’ GO and proteome analyst categories

| Contig | Annotation | Uniprot Accession |
| --- | --- | --- |
|  | **‘Cytosol’** |  |
| Sm04870 | Proteasome activator PA28 subunit | Q86BY1 |
| Sm00525 | Proteasome subunit N3 | Q90ZQ5 |
| Sm04498 | Proteasome subunit beta type 1-B | Q9IB83 |
| Sm00746 | Ubiquitin interaction motif | O17453 |
| Sm01713 | 26S protease regulatory subunit 8 | P62198 |
| Sm05291 | 26S protease regulatory subunit 6A | O14126 |
| Sm03179 | Proteasome regulatory particle | Q9GZH5 |
|  | **‘Golgi’** |  |
| Sm01118 | Transmembrane trafficking protein | Q6P227 |
| Sm01225 | putative Amino acid transporter | Q9GKM3 |
| Sm00144 | Sodium / potassium ATPase beta chain | Q7Q3B0 |
| Sm01205 | putative Ras-related protein Rab-6 homolog | P34213 |
| Sm11682 | Rab family protein 1 | Q86ET1 |
| Sm04318 | putative Beta-1,4-mannosyltransferase | Q7SXK9 |
| Sm04855 | putative Alpha-6-fucosyltransferase | Q6EV75 |
| Sm05164 | Clathrin adaptor complex small chain | Q6XI64 |
| Sm08758 | Coatomer beta' subunit | Q7ZTR0 |
| Sm11213 | Ras-related protein Rab-14 | Q86EY7 |
| Sm06649 | transmemberane protein, putative | Q9XUY5 |
| Sm03589 | ADP-ribosylation factor, arf, putative | Q86E39 |
| Sm13030 | putative Syntaxin-like protein | Q8T0Z9 |
| Sm03904 | putative Ectonucleotide pyrophosphatase/phosphodiesterase 5 precursor | Q9UJA9 |
| Sm09044 | putative Conserved oligomeric Golgi complex component 4 | Q8R1U1 |
| Sm12438 | Hypothetical GABA(A) receptor-associated protein like-2 | Q86LP9 |
| Sm04669 | Mannosyl-oligosaccharide 1,2-alpha-mannosidase IB | Q6DFL1 |
| Sm12751 | HGF-regulated tyrosine kinase substrate | Q6P7F6 |
| Sm09515 | putative 3-beta-glucuronosyltransferase | Q7YXB1 |
|  | **‘GTP binding’** |  |
| Sm01001 | Rac GTPase | Q8I898 |
| Sm00509 | Rab-related GTP-binding protein | Q26554 |
| Sm29703 | Probable Ras-related protein Rab-4A | Q9GP33 |
| Sm01205 | Ras-related protein Rab-6 homolog F59B2.7 | P34213 |
| Sm11682 | Ras | Q86ET1 |
| Sm02151 | GTP-binding signal recognition particle | Q7PSJ1 |
| Sm06624 | Beta-tubulin | Q7Z1I4 |
| Sm04550 | Ras-related protein Rap-1b | Q99JI6 |
| Sm06722 | Rac GTPase | Q8I898 |
| Sm09228 | Ras | Q86ES9 |
| Sm13217 | Ras related small G protein RAL-A (RALA protein) | Q9I8H8 |
| Sm04990 | Arf | Q86F33 |
| Sm13242 | Elongation factor G (EF-G) | Q8DI43 |
| Sm01350 | Rab4A-like protein | O44213 |
| Sm03786 | Ras-related protein Rab-11A (Rab-11) | P62493 |
| Sm01686 | Elongation factor 1-alpha | Q94747 |
| Sm00654 | Alpha tubulin | Q26595 |
| Sm11213 | Ras-related protein Rab-14 | Q86EY7 |
| Sm01081 | Initiation factor 2B | Q86E66 |
| Sm06260 | Guanine nucleotide-binding protein G | P30669 |
| Sm03589 | ADP-ribosylation factor, arf, putative | Q86E39 |
|  | **‘Small GTPase-mediated signal transduction’** |  |
| Sm01001 | Rac GTPase | Q8I898 |
| Sm00509 | Rab-related GTP-binding protein | Q26554 |
| Sm29703 | Probable Ras-related protein Rab-4A | Q9GP33 |
| Sm01205 | Ras-related protein Rab-6 homolog F59B2.7 | P34213 |
| Sm11682 | Rab family protein 1 | Q86ET1 |
| Sm04550 | Ras-related protein Rap-1b (GTP-binding protein smg p21B) | Q99JI6 |
| Sm06722 | Rac GTPase | Q8I898 |
| Sm09228 | Ras | Q86ES9 |
| Sm13217 | Ras-related small G protein RAL-A (RALA protein) | Q9I8H8 |
| Sm04990 | ADP-ribosylation factor | Q86F33 |
| Sm01350 | Rab4A-like protein | O44213 |
| Sm03786 | Ras-related protein Rab-11A (Rab-11) | P62493 |
| Sm11213 | Ras-related protein Rab-14 | Q86EY7 |
| Sm03589 | ADP-ribosylation factor, arf, putative | Q86E39 |
