# Supplementary figures and images for: Altered Patterns of Gene Expression Underlying the Enhanced Immunogenicity of Radiation-Attenuated Schistosomes
Source: PLoS Negl Trop Dis. 2008 May 21;2(5):e240. doi: 10.1371/journal.pntd.0000240 (PMC2375114; doi:10.1371/journal.pntd.0000240)

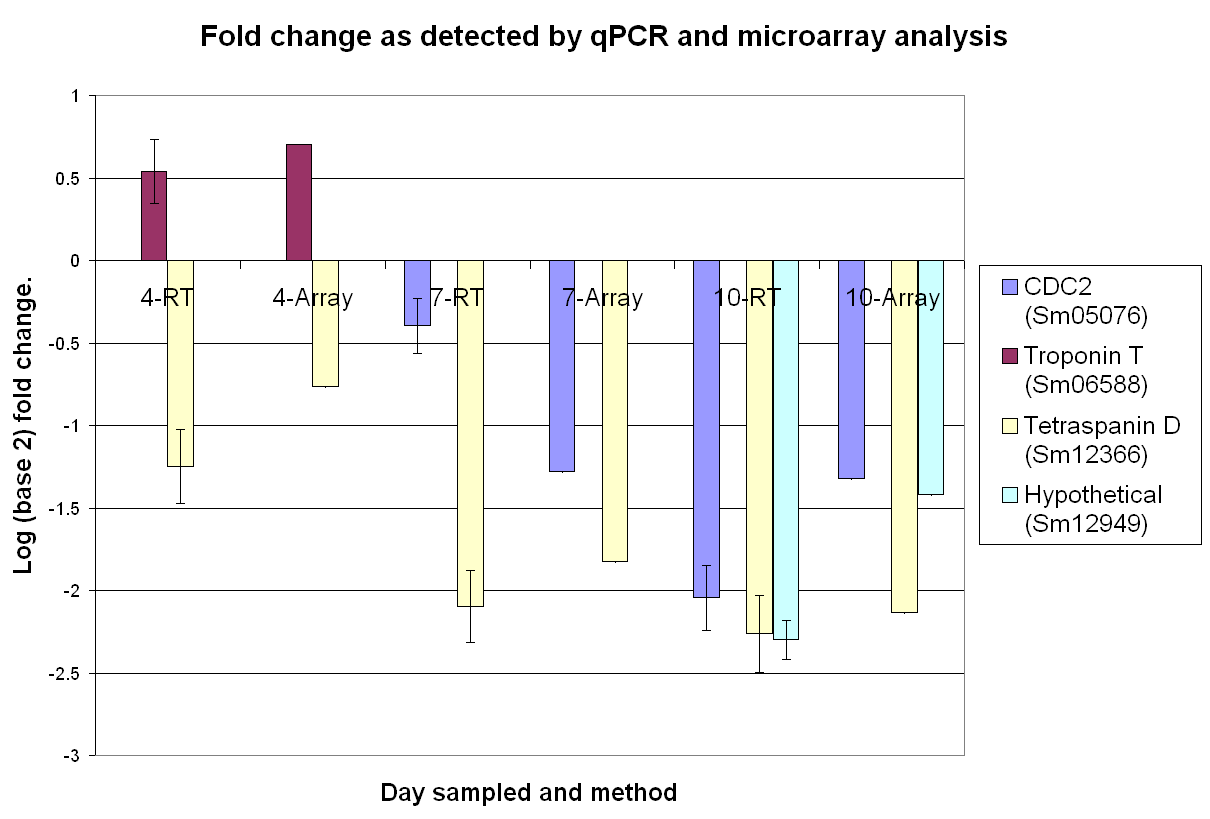

Supplement: Figure S1 — To validate array data qPCR was performed on four representative genes for comparison. The bar chart illustrates the fold differences determined by the two methods. Only qPCR produces means +/− SEM. (3.00 MB TIF) [file pntd.0000240.s006.tif]
